# Knowledge, Attitudes, and Practices Toward Coronavirus Disease 2019 in the Central Area of Iran: A Population-Based Study

**DOI:** 10.3389/fpubh.2020.599007

**Published:** 2020-12-08

**Authors:** Rahmatollah Moradzadeh, Javad Nazari, Mohsen Shamsi, Saeed Amini

**Affiliations:** ^1^Department of Epidemiology, School of Health, Arak University of Medical Sciences, Arak, Iran; ^2^Department of Pediatrics, School of Medicine, Arak University of Medical Sciences, Arak, Iran; ^3^Department of Health Education and Health Promotion, Faculty of Health, Arak University of Medical Sciences, Arak, Iran; ^4^Department of Health Management, School of Health, Arak University of Medical Sciences, Arak, Iran

**Keywords:** knowledge, attitudes, practices, COVID-19, Iran

## Abstract

**Background:** The spread of the coronavirus disease 2019 (COVID-19) pandemic has imposed high threats on global health, life and work style, and social and economic development. The current study aimed to extract knowledge, attitudes, and practices related to COVID-19 among the general population in the central area of Iran.

**Method:** A cross-sectional study was conducted in Arak City between April and May 2020. Stratified random sampling was applied to select the study participants. Phone interview was applied to collect the data. Data were collected using a questionnaire that was constructed and validated in this study. The questionnaire included demographic variables and items about knowledge, attitudes, and practices toward COVID-19. Descriptive and inferential analyses were conducted in STATA software.

**Results:** In total, 544 participants completed the questionnaire; 76% of the participants accounted COVID-19 as a high threat 1 month from the onset of COVID-19. From the maximum attainable scores of 1, 6, and 6, for COVID-19-related knowledge, attitudes, and practices, means of 0.77 (0.13), 4.97 (0.63), and 5.35 (0.70) were obtained, respectively. Females had a higher practice score (5.4 ± 0.6). The participants with a family history of heart and respiratory diseases had significantly higher attitude and practice scores. SMS from the Ministry of Health had a significant impact on knowledge, attitude, and practice scores (*p* < 0.05).

**Conclusion:** Higher attention should be given to increase the knowledge, attitudes, and practices of men and the housewife group. COVID-19 preventive messaging from the Ministry of Health was among the most influential methods of increasing knowledge that attracted public attention.

## Introduction

An emerging highly infectious disease with the main symptoms of dry cough, dyspnea, and fever called coronavirus disease 2019 (COVID-19) was first diagnosed in December 2019 in Wuhan City, China (1). Its ongoing spread, across countries and continents, has evoked the World Health Organization (WHO) to declare it as an international public health emergency on January 30 and requested collaborative efforts of all countries to contain its rapid spread (2). Regarding Iran, the first cases were reported from Qom Province on February 19, 2020, and then, several days later, in Markazi Province, as the second place, the location where the current study was performed (3–5).

The spread of COVID-19 has imposed high threats on global health, life and work style, and social and economic development (6, 7). To face this threat, countries have issued many guidelines on different aspects of prevention and control of COVID-19 (8). These guidelines are not effective unless the knowledge, attitudes, and practices of the public are improved. Lessons learned from the severe acute respiratory syndrome (SARS) epidemic point out that high knowledge, attitudes, and practices (KAP) toward different epidemics decrease stress and panic and pave the way toward their prevention and control (9, 10). Also, high public awareness toward Ebola and the Middle East respiratory syndrome (MERS) provided the background to control them (11, 12).

To plan and design effective and universal healthcare packages on COVID-19, it is necessary to extract KAP of the public, which this study aimed to perform. This study indicated the shortcomings and gaps between what COVID-19 measures have been performed and what needs to be performed in terms of improving public KAP.

As Arak city was among the first cities that have reported cases of COVID-19 (7) and also due to its geographical position that connects many provinces to each other (7, 13), the city can be introduced as one of the main COVID-19 epicenters in Iran. On the other hand, understanding the public KAP toward COVID-19 facilitates its management and control. So, the current study was performed to extract knowledge, attitudes, and practices related to COVID-19 among the general population in the central area of Iran.

## Methods

This is a cross-sectional study conducted in Arak City, Iran. Arak is the capital of Markazi Province in the center of Iran with an estimated population of nearly 600,000 people (13). The study was conducted between April 11 and May 2, 2020. The source population for this study was all the female and male population living in Arak City.

In order to obtain a representative sample, stratified random sampling was applied to select the study participants from the source population. First, the population was divided into 50 strata, based on the centers providing health services. From each stratum, the participants were selected by simple random sampling based on the proportion and size of each center. All people older than 18 years old were eligible for the study.

A phone interview was applied by 10 health providers to collect the data. The phone number list was extracted from the SIB system (in Persian: Samane Yekparcheh Behdasht or Integrated Health Record System), where the household's characteristics of each stratum are registered by the centers. Interviewers attended a training session before collecting the data. The SIB system is a platform that provides the most comprehensive electronic health records on personal demographic information, records of diseases, medical records, and all information affecting individual health. The interesting point about this system is that it has interactions with other systems aside from the Ministry of Health and Medical Education such as the Insurance System, Forensic Medicine, etc. This system has been established in more than 36,000 urban and rural regions around the country, employing more than 130,190 healthcare staff including Behvarz (primary healthcare providers), midwife, nurse, mental health expert, general physician, specialist, general dentist, etc. (14).

The proportion formula of $Z_{1-\frac{\alpha }{2}}^{2}\times p\left(1-p\right)/precision^{2}$ was used to estimate minimum sample size [with estimated knowledge level 94% (15), 2% precision, 95% confidence level, and considering 10% nonresponse proportion], so the sample size obtained was 600. The total number of participants who answered the questionnaire was 544, making the response rate of 91%.

Data were collected using the questionnaire constructed and validated in this study. In this regard, based on the literature and guidelines issued by the WHO (16), items related to KAP of COVID-19 were included. The content validity of the instrument was assessed by 10 experts in the fields of epidemiology, health education, and infectious diseases (with respect to simplicity, relativity, and importance). Then, internal consistency was assessed among 30 people by Cronbach's alpha. The questionnaire's validity and reliability were approved; Cronbach's alpha values for knowledge, attitude, and practice domains were 0.98, 0.95, and 0.91, respectively.

The study variables included age (years); sex (female/male); status (single/married); education (illiterate/elementary/intermediate/diploma/academic); job (housewife/office employee/working/others); family history of heart, blood pressure, and respiratory diseases (yes/no); accounting COVID-19 as a threat (not at all/very low/low/relatively/high/very high); and source of acquired information (TV and radio/family/friends/health staff/internet/SMS from the Ministry of Health/pamphlets and posters/others).

The knowledge section of the questionnaire with 17 items had “yes,” “no,” or “I don't know” answers. Correct and incorrect answers scored 1 and 0, respectively. The attitude section had 14 items, and the answers were included on a six-point Likert scale (1, strongly disagree; 2, moderately disagree; 3, slightly disagree; 4, slightly agree; 5, moderately agree; 6, strongly agree). The practice section had 15 items and the answers were included on a six-point Likert scale (1, never; 2, very rarely; 3, rarely; 4, sometimes; 5, usually; 6, always). The items related to KAP of COVID-19 are presented in Tables 1–3.

**Table 1:** The percentage of correct answers for knowledge items of the KAP questionnaire among the participants.

**Knowledge items**	**Correct answer (%)**
Do asymptomatic people transmit the disease to others?	77
Does arbitrary treatment of COVID-19 with different drugs useful?	77
Does washing hands with soap and water help prevent the disease?	96
Are people with chronic background diseases (such as cardiopulmonary diseases) considered as high-risk groups for coronary heart disease?	94
Can the correct use of mask in patients reduce the transmission of the disease?	95
Is COVID-19 transmitted through coughing and sneezing?	97
Can the disease be transmitted between humans and animals?	70
Can the disease be transmitted through well-cooked products?	54
Does going to crowded places increase the risk of infection?	95
Does touching elevator keys or bank passers-by transmit the disease?	95
Does staying home, and not leaving home as much as possible, have an effect on reducing the transmission?	95
In your opinion, do you have enough knowledge about COVID-19?	74
How long is asymptomatic time period for COVID-19?	60
How deadly can this disease be?	39
How often should a regular mask be replaced?	67
What part of the body is affected by this disease?	84

**Table 2:** The percentage of attitude items for the KAP questionnaire among the participants.

**Items**	Strongly agree^*^N (%)
I'm worried about myself or a family member getting the disease.	173 (33)
I believe that the transfer of COVID-19 can be prevented by following national or international guidelines.	146 (28)
I believe that getting information about COVID-19 through social networks is enough.	60 (12)
I believe that wearing masks in crowded places is effective.	225 (43)
I believe that hand disinfection is effective in reducing disease transmission.	284 (55)
I believe that disinfecting objects is effective in reducing COVID-19 transmission.	254 (50)
I believe that COVID-19 can be treated at home.	113 (22)
I believe that washing hands with soap and water helps prevent the disease.	278 (53)
I believe that the correct use of mask can reduce the transmission of the disease to others.	238 (46)
I believe that the correct use of mask can reduce the transmission of the disease to a person.	210 (41)
I believe that mask should be changed every few hours.	215 (42)
I believe that staying home, and not leaving home as much as possible, has an effect on reducing the transmission.	293 (56)
I believe that touching the elevator or ATM keys is effective in the disease transmission.	301 (57)
I believe that going to crowded places increases the risk of infection.	348 (66)

* *Other points are not indicated*.

**Table 3:** The percentage of practice items of the KAP questionnaire among the study participants.

**Items**	Always^*^N (%)
I avoid crowded places and gatherings as much as possible.	291 (55)
I avoid entering closed places as much as possible.	258 (50)
I avoid raw foods as much as possible.	292 (56)
I use mask as much as possible in crowded places.	280 (54)
I cover my mouth and nose when I sneeze and cough.	322 (61)
I avoid touching my nose, mouth, and eyes as much as possible.	292 (56)
I always throw the used mask in the trash.	310 (60)
I avoid shaking hands with others as much as possible.	341 (65)
I disinfect objects as much as possible.	291 (56)
As much as possible, I recommend that a person with the disease symptoms to see a doctor.	291 (56)
I wash my hands with soap and water several times a day as much as possible.	337 (64)
I do not get close to another person as much as possible.	285 (54)
I change mask every few hours as much as possible.	256 (49)
I do not touch the elevator and ATM keys without protection as much as possible.	309 (58)

* *Other points are not indicated*.

### Statistical Analysis

Descriptive and inferential statistics were applied by using STATA 12.0. Independent sample *t* test and one-way analysis of variance were applied to present differences in KAP of the population by demographic characteristics. Spearman correlation test was used to identify any correlation between knowledge, attitudes, and practices. *p* < 0.05 was considered in the all tests to indicate significance.

## Results

### Descriptive Findings

In total, 544 participants completed the questionnaires. The nonresponse rate was 9% (*n* = 56) and these were the ones who did not answer the phone call. Mean age (SD) was 36 (10) years old. Females were 69% (*N* = 376) of the participants. Descriptive findings of other demographic characteristic are indicated in Table 4. Furthermore, 78% of the participants did not report any family history of heart and respiratory diseases. During 1 month from the onset of the COVID-19 epidemic, accounting COVID-19 as a high and very high threat was reported by 76% of the participants (Table 4). The most reported source of acquiring information about COVID-19 was TV and radio (86%) (Table 5). In total, from the maximum attainable scores equal to 1, 6, and 6, for COVID-19-related knowledge, attitudes, and practices, means (SDs) of 0.77 (0.13), 4.97 (0.63), and 5.35 (0.70) were obtained, respectively.

**Table 4:** Descriptive characteristics and comparison of COVID-19-related knowledge, attitudes, and practices in Arak in 2020.

**Variable**		***N* (%)**	**Knowledge**		**Attitudes**		**Practices**	
			**Mean (SD)**	***p* value**	**Mean (SD)**	***p* value**	**Mean (SD)**	***p* value**
Total	Mean (SD), min, max	544	0.77 (0.13), 0, 1	–	4.97 (0.63), 2, 6	–	5.35 (0.70), 2.3, 6	–
Sex	Male	167 (31)	0.79 (0.11)	0.13	4.94 (0.7)	0.49	5.2 (0.8)	0.02
	Female	376 (69)	0.77 (0.13)		4.98 (0.6)		5.4 (0.6)	
Age	Mean (SD), (min–max)	36 (10), (18–89)	*r* = −0.03	0.38	*r* = 0.03	0.45	*r* = −0.04	0.34
	0–25	53 (11)	ANOVA	0.38		0.97		0.67
	25–35	234 (47)						
	35–45	143 (28)						
	45–55	43 (9)						
	55–90	27 (5)						
Status	Married	489 (90)	0.77 (0.13)	0.1	4.97 (0.6)	0.84	5.35 (0.7)	0.9
	Single	53 (10)	0.8 (0.15)		4.95 (0.9)		5.33 (0.8)	
Education	Illiterate	7 (1)	*r* = 0.29	0.001	*r* = 0.08	0.09	*r* = 0.07	0.09
	Elementary	19 (4)	Spearman					
	Intermediate	67 (13)						
	Diploma	192 (36)						
	Academic	241 (46)						
Job	Housewife	260 (53)	0.75 (0.1)	0.001	4.97 (0.6)	0.97	5.38 (0.7)	0.06
	Office employee	107 (22)	0.84 (0.1)		4.97 (0.6)		5.40 (0.6)	
	Working	13 (3)	0.76 (0.1)		4.99 (0.7)		5.07 (0.9)	
	Others	109 (22)	0.77 (0.1)		4.94 (0.7)		5.20 (0.8)	
Family history of disease^1^	Yes	114 (22)	0.79 (0.1)	0.22	5.10 (0.5)	0.01	5.50 (0.6)	0.02
	No	414 (78)	0.77 (0.1)		4.94 (0.6)		5.31 (0.7)	
Accounting COVID-19 as a threat	Not at all	10 (2)						
	Very low	8 (1)	*r* = 0.17	0.001	*r* = 0.31	0.001	*r* = 0.30	0.001
	Low	18 (3)	Spearman					
	Relatively	93 (18)						
	High	211 (40)						
	Very high	189 (36)						

1 *Include heart, blood pressure, and respiratory diseases*.

**Table 5:** Characteristics of the participants by source of acquired information in Arak in 2020.

**Source of acquired information**		***N* (%)**	**Knowledge**		**Attitudes**		**Practices**	
			**Mean (SD)**	***p* value**	**Mean (SD)**	***p* value**	**Mean (SD)**	***p* value**
TV and radio	Yes	422 (86)	0.78 (0.11)	0.6	5.0 (0.6)	0.04	5.4 (0.7)	0.01
	No	67 (14)	0.77 (0.14)		4.8 (0.7)		5.2 (0.8)	
Family	Yes	78 (16)	0.79 (0.1)	0.3	5.2 (0.5)	0.006	5.44 (0.6)	0.3
	No	410 (84)	0.78 (0.1)		4.9 (0.6)		5.36 (0.7)	
Friends	Yes	58 (12)	0.79 (0.1)	0.4	5.1 (0.5)	0.4	5.32 (0.7)	0.6
	No	430 (88)	0.78 (0.1)		5.0 (0.6)		5.38 (0.7)	
Health staff	Yes	220 (45)	0.79 (0.1)	0.009	5.02 (0.6)	0.3	5.38 (0.7)	0.7
	No	268 (55)	0.77 (0.1)		4.95 (0.6)		5.36 (0.6)	
Internet	Yes	187 (38)	0.82 (0.09)	0.001	5.06 (0.5)	0.02	5.43 (0.6)	0.15
	No	301 (62)	0.75 (0.1)		4.93 (0.7)		5.33 (0.7)	
SMS from the Ministry of Health	Yes	210 (43)	0.81 (0.1)	0.001	5.06 (0.6)	0.02	5.44 (0.6)	0.047
	No	278 (57)	0.76 (0.1)		4.92 (0.6)		5.32 (0.7)	
Pamphlets and posters	Yes	61 (12)	0.80 (0.1)	0.15	5.13 (0.4)	0.04	5.45 (0.6)	0.3
	No	427 (88)	0.78 (0.1)		4.96 (0.6)		5.36 (0.7)	
Others	Yes	17 (3)	0.79 (0.1)	0.7	4.84 (0.7)	0.3	5.17 (0.5)	0.2
	No	471 (97)	0.78 (0.1)		4.99 (0.6)		5.38 (0.7)	

### Analytical Findings

The results showed that females had a statistically significant higher practice score (5.4 ± 0.6) compared with males (5.2 ± 0.8); however, there was no significant difference between them for knowledge and attitudes. There were no differences in means of knowledge, attitude, and practice scores based on age and status of the participants. In addition, there was no statistically significant linear correlation between age with knowledge, attitudes, and practices. There was a weak positive correlation between knowledge and years of education, which was statistically significant (*r* = 0.29, *p* = 0.001), but there was no correlation between attitudes and practices with education. There was a statistically significant difference between job groups (*p* = 0.001) in acquired knowledge score. In addition, there was a statistically nonsignificant difference between job groups in the acquired attitude (*p* = 0.97) and practice (*p* = 0.06) scores. The results showed that participants with a family history of heart and respiratory diseases had significantly higher attitude (*p* = 0.01) and practice (*p* = 0.02) scores statistically compared with participants without a family history of heart and respiratory diseases. However, there was no statistically significant difference in the knowledge of the two groups (*p* = 0.22). There was a weak statistically significant positive correlation between knowledge (*p* = 0.001), attitude (*p* = 0.001), and practice (*p* = 0.001) scores in terms of accounting COVID-19 as a threat (Table 4).

There was a statistically significant difference in the means of knowledge scores for health staff, internet, and SMS from the Ministry of Health; attitude scores for TV and radio, family, internet, SMS from the Ministry of Health, pamphlets, and posters; and practice scores for TV and radio and SMS from the Ministry of Health as the acquired source of information (*p* < 0.05) (Table 5).

## Discussion

Due to high morbidity and mortality and the lack of a specific and effective treatment or vaccine, preventive measures and increasing public KAP play a unique role in confronting COVID-19. So, this study was performed to analyze these measures in one of the first Iranian provinces where COVID-19 cases were reported (7).

This study indicated that there was no significant relationship between the age of the participants and COVID-19 KAP. The published studies have reported contradictory results. Accordingly, the study of Al-Hanawi et al. on COVID-19 KAP among the public in Saudi Arabia indicated that older people have higher knowledge toward COVID-19 (17). In sum, it has been proven that age has a direct link with knowledge level (18). The reason behind the lack of relationship between age and COVID-19 KAP in the current study needs further studies. However, young people having higher access to social media and older people having higher experience in the face of diseases including COVID-19 have neutralized the effect of age on COVID-19 KAP.

The results indicated that although there is no difference in terms of knowledge and attitudes between men and women, women have higher COVID-19 practice scores than men. This means that men despite having the required knowledge and attitude perform lower preventive measures against COVID-19. This necessitates specific designs and programs targeting this group. The study of Bao-Liang Zhong et al. on Chinese residents indicated that women have good knowledge, optimistic attitudes, and appropriate practices toward COVID-19 (19). Another study by Chen Yan et al. stated that men adhere less toward wearing masks and hand washing than women (20), suggesting that more focus should be given to men in designing preventive and educational programs.

This study indicated that although those with higher education level have higher knowledge related to COVID-19, it is not generalized to attitude and practice status. In other words, there was no significant difference between attitudes and practices toward COVID-19 among the participants with illiterate, elementary, and university literacy level.

The results indicated that the office employee group has the highest knowledge regarding COVID-19. The study of Rahman and Sathi on Bangladeshi internet users indicated that the likelihood of staying home and wearing mask increases by each unit increase in the knowledge score (21). This indicates the importance of improving knowledge status as the first step of developing healthy behaviors. The study of Azlan et al. on KAP of the general population showed that those working in the public sector have the highest positive attitude toward COVID-19. They described that this may be due to their affiliation to the government that leads to report a high positive attitude in this regard (22). However, the high knowledge of the employee group in this study may be due to workplace education, guidelines issued by the Ministry of Health for workers, and their higher literacy level than the public.

TV and radio were the media most frequently used by the participants to obtain information about COVID-19. The KAP study of Italian students toward COVID-19 by Souli and Dilucca showed that the most frequently reported media were television and then Facebook, WhatsApp, and Instagram (23). The participants reported that different media including TV and radio, Ministry of Health messaging, pamphlets and posters, internet, and social media are effective on improving their knowledge, attitudes, and practices toward COVID-19. On the other hand, Chen Yan et al. stated that social media such as micro letter and QQ have replaced TV and radio and people no longer acquire their information through these traditional media (20). However, it does not matter how COVID-19 information is obtained; the most important issue is supervising the media to provide reliable and relevant scientific information in a timely manner.

### Study Limitations

This is among the first studies performed to extract the KAP of the general public toward COVID-19 in the central area of Iran, which is useful in designing interventional packages to control it. Although the questioners were educated to supervise on completing the questions, and also the importance of honesty and correct response by participants was emphasized, one of the issues threatening self-reporting studies is reporting bias that needs to be improved using administrative data in future studies.

## Conclusions

The present study provided a comprehensive assessment of KAP of the general population. The results indicated that women have better COVID-19-related practices, education level is effective on COVID-19 knowledge, and TV and radio are the main sources of acquiring information. This study indicated that higher attention should be given to increase men's and the housewife group's KAP. COVID-19 preventive messaging from the Ministry of Health was among the most influential methods of increasing knowledge that attracted public attention.

## Data Availability Statement

The raw data supporting the conclusions of this article will be made available by the authors, without undue reservation.

## Ethics Statement

The studies involving human participants were reviewed and approved by an oral and written informed consent was obtained from each participant prior to the interview. This study has been approved ethically by Research and Technology Deputy of Arak University of Medical Sciences (Ethical Code Number: IR.ARAKMU.REC.1398.328). The patients/participants provided their written informed consent to participate in this study.

## Author Contributions

RM and SA designed the study, performed the statistical analysis, and drafted the manuscript. JN and MS revised the manuscript critically for important intellectual content and gave final approval of the manuscript. RM, SA, JN, and MS contributed to the conception and design of data. All authors contributed to the article and approved the submitted version.

## Conflict of Interest

The authors declare that the research was conducted in the absence of any commercial or financial relationships that could be construed as a potential conflict of interest.

## Abbreviations

WHO: world health organization
SARS: severe acute respiratory syndrome
MERS: middle east respiratory syndrome
KAP: knowledge, attitudes, and practices.
